# Nodal tumor volume as a prognostic factor for oral squamous cell carcinoma—a systematic review

**DOI:** 10.3389/froh.2023.1229931

**Published:** 2023-08-16

**Authors:** Mischa Bernasconi, Ante Bilic, Martin Kauke-Navarro, Ali-Farid Safi

**Affiliations:** ^1^ Faculty of Medicine, University of Berne, Berne, Switzerland; ^2^ Division of Plastic Surgery, Department of Surgery, Yale New Haven Hospital, Yale School of Medicine, New Haven, CT, United States; ^3^ Craniologicum, Center for Craniomaxillofacial Surgery, Berne, Switzerland

**Keywords:** oral squamous cell carcinoma, lymph node, volume, prognostic factor, nodal metastasis

## Abstract

Oral squamous cell carcinoma (OSCC) is a complex disease with a high potential for lymph node metastasis and poor survival rates. Accurate nodal staging is crucial for prognostic assessment and treatment planning in OSCC. Recent research has suggested that nodal tumor volume (NTV) may be a more accurate indicator of nodal disease burden than traditional staging methods. However, the prognostic significance of NTV in OSCC remains unclear. This systematic review aims to evaluate the existing evidence on the relationship between NTV and prognosis in OSCC. A comprehensive search of electronic databases was conducted, and studies meeting inclusion criteria were critically appraised and synthesized. Our review identified 23 studies that investigated the prognostic significance of NTV in OSCC. The majority of studies reported that larger NTV was associated with poorer survival outcomes, although the strength of the association varied. The review also identified several areas for future research, including the standardization of NTV measurement and the integration of NTV into the broader landscape of OSCC management. In conclusion, our review suggests that NTV holds promise as a novel prognostic factor in OSCC, but more research is needed to fully elucidate its potential and inform clinical decision-making.

## 1. Introduction

Oral squamous cell carcinoma (OSCC) is a common and aggressive form of head and neck cancer, characterized by local invasion and a high likelihood of lymph node metastasis. OSCC accounts for over 90% of oral malignancies. Mortality rates for oral cancer remain around 50% in most countries, with no significant improvements observed over the last few decades (1–3). For patients diagnosed with metastatic disease, the 5-year relative survival rate is between 29.5%–32.5% (4).

Over 50% of oral squamous cell carcinoma (OSCC) cases occur in males during their 6th and 7th decades, though the trend for oral cancer to affect younger individuals—those under the age of 45—is increasingly apparent (5). In patients diagnosed with OSCC, staging according to the TNM classification system of Union International Contre le Cancer (UICC)/American Joint Committee in Cancer (AJCC) plays a critical role in diagnostic evaluation and therapeutic decision-making (6). The presence of cervical lymph node metastases is a crucial prognostic factor for patients with OSCC. Current nodal staging (N staging) systems primarily focus on the number, size, and laterality of involved lymph nodes (1–3). However, these systems may not fully capture the complexity of nodal disease. Emerging evidence suggests that nodal tumor volume (NTV), which accounts for the three-dimensional nature of the tumor within the lymph node, might provide a more nuanced understanding of disease progression and more accurately predict patient outcomes (3, 7).

Recent technological advancements in threedimensional imaging analysis [based for example on computed tomography (CT) or magnetic resonance imaging (MRI)] offer new possibilities for approximating volumetric changes. CT provides high-resolution images that enable multiplanar reconstructions in a short scanning time, while MRI supplies higher soft tissue contrast and with functional pulse sequences, more detailed information about lesions can be obtained (8–10).

Accurate evaluation of NTV requires precise segmentation of tumor-containing lymph nodes from imaging data. Several methods are available for this task, which can broadly be divided into manual, semi-automatic, and fully automatic methods.

Manual segmentation is the traditional method and is still widely used in clinical practice. In this method, an expert radiologist manually delineates the tumor boundaries on each slice of the imaging data, typically using a graphical user interface. While this method can provide highly accurate results due to the involvement of expert knowledge, it is time-consuming and subject to inter–and intra-observer variability. Semi-automatic segmentation methods aim to reduce the time required for segmentation and improve consistency, while still leveraging expert knowledge. These methods typically require the user to provide some input, such as selecting seed points within the tumor, after which the algorithm expands the segmentation based on pre-defined criteria. Fully automatic segmentation methods aim to further reduce the time required for segmentation and eliminate observer bias. These methods often involve machine learning algorithms, which learn to recognize and delineate tumors based on large datasets of previously segmented images. Fully automatic methods have the potential to provide highly accurate and consistent segmentations, but their performance depends on the quality and representativeness of the training dataSeveral studies have emphasized the prognostic value of metastatic nodal tumor volume in patients with head and neck squamous cell carcinoma (HNSCC). A systematic review by Moumoulidis et al. (11) concluded that the nodal volume could serve as an imaging biomarker to predict diverse clinical outcomes in HNSCC patients (11). However, this review did not discuss the specific prognostic impact of metastatic nodal tumor volume in patients with OSCC. As such, it is critical to evaluate OSCC as an independent entity, given its status as the most common head and neck cancer, and its biological behavior, which differs significantly in terms of local aggressiveness and metastatic capacities (12).

## 2. Methods

Due to the objective of this review—identify the prognostic factor of nodal volume in patients with OSCC—the eligibility criteria for included studies were following: (1) studies which included neoplasms in the oral cavity (2) Studies investigating the association between nodal tumor volume and clinical outcome (3) Studies measuring the separate tumor and nodal metastases volume before any treatment has taken place.

The exclusion criteria were the following: (1) no separate nodal volume or separate primary tumor sites analysis 2 Use of other radiographic parameters or ultrasound (3) Not original research studies, case reports, editorials, commentaries.

The search for articles was conducted on the bibliographic Database PubMed and the review was performed by following the PRISMA (preferred reporting items for systematic reviews and meta-analyses) statement (13).

For the search of the relevant published studies the keywords were taken from the systematic review mentioned above:

(((((((((((volum*) OR “Lymph Nodes/diagnostic imaging” [Mesh])) AND ((((“Head and Neck Neoplasms” [Mesh]) OR “Squamous Cell Carcinoma of Head and Neck” [Mesh])) OR hypopharyngeal))) NOT esopha*) NOT thyroid) NOT parathyroid) NOT sinonasal) NOT melanoma) NOT gland) NOT nasopharyn*.

Studies examining nasopharyngeal and sinonasal carcinomas were excluded because of differences in entity, pathology and progression. Case reports, systematic reviews and meta-analyses were also excluded.

The data were extracted by the two authors (MB, AS) and merged directly into table. The quality assessment of the retrieved studies was performed by two authors (MB, AS) considering the effective public health practice project (EPHPP) assessment tool for quantitative studies (14).

## 3. Results

**Figure 1:**
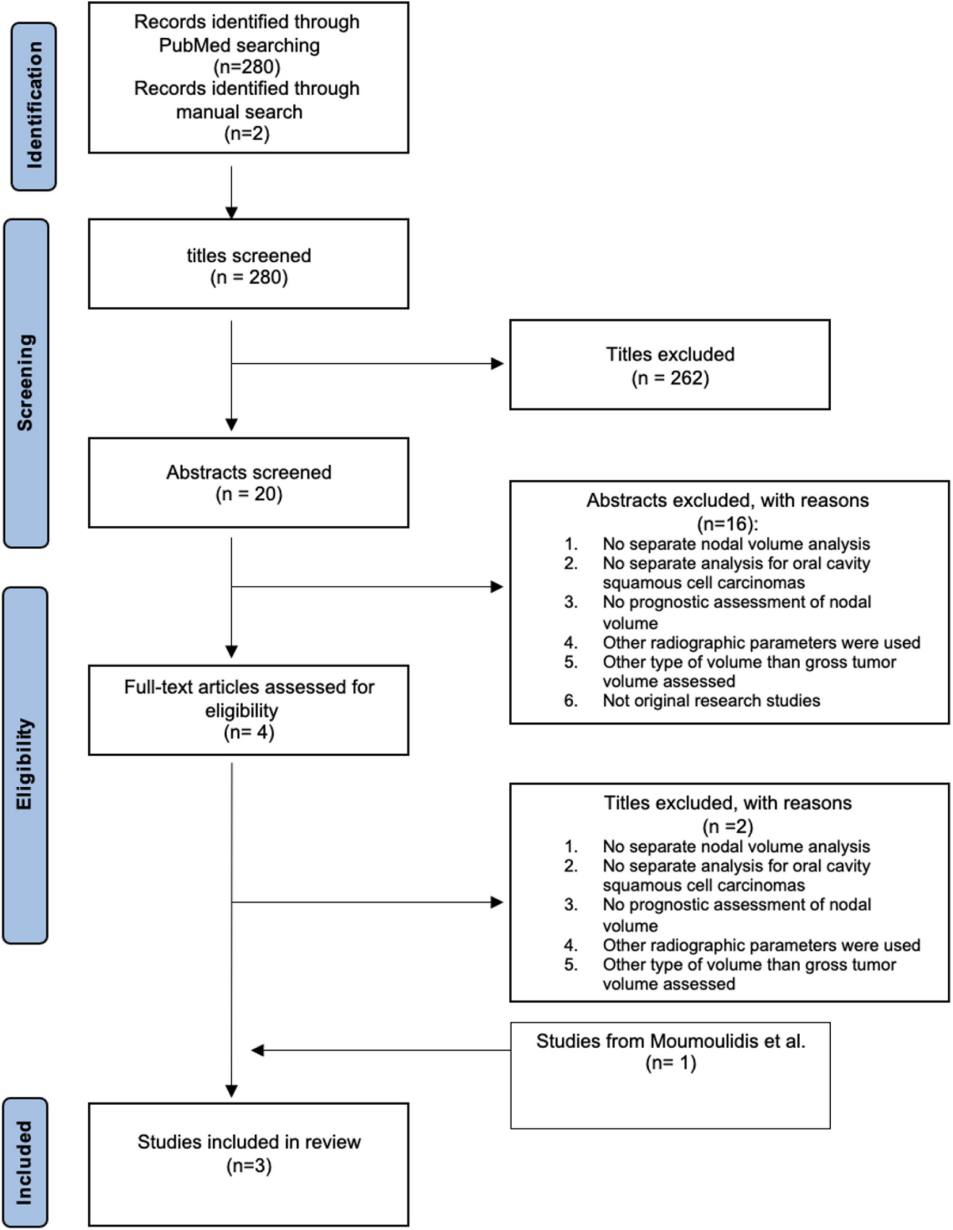
PRISMA process flow diagram for study selection.

Overall, 280 records were retrieved from PubMed (from February 2021 up to February 2022) and 2 studies were identified through manual search. After initial screening 262 titles and 16 abstracts were excluded due to reasons listed in Figure 1. A total of 4 full-text articles were assessed for eligibility whereby 2 titles were excluded based on strict exclusion criteria. One study (Safi et al.) (3) is acquired from the included studies of the systematic review by Moumoulidis et al. (11). Further, two studies (Ljumanovic et al. (15) Vergeer et al. (16)) were identified by manual scanning the references of the decisive studies in the systematic review (Figure 1).

In summary, this results in a number of 490 patients with OSCC. Table 1 summarizes the basic characteristics of the included studies as they are: first author's surname, study type and year of publication; primary sites of the tumor and staging; imaging technique and type of assessed volume; the treatment performed; the number of patients; follow-up period; treatment outcome and survival statistic with the corresponding 95% confidence interval (CI).

**Table 1:** Main characteristics of the eligible studies included in the systematic review.

First author, year, study type	Primary site and staging	Imaging technique/volume type	Treatment	Number of patients	Follow-up period	Treatment outcome, survival statistic
Safi et al. (3) Retrospective	OCSCC; Subsites: nd	CT	Neck dissection (Level I to V) and radiotherapy in locally advanced disease	*N* = 100	>3 months	LR
	Stages III/IV (T4b, N2c and N3 excluded)	NV				HR: 20.926 (4.824–90.774), *p *= <0.001
Vergeer et al. (16) Retrospective	HNSCC; Subsites: oral cavity, Oropharynx, Hypopharynx, Larynx, other	CT	Primary radiotherapy or chemotherapy	Total *N* = 79;OSCC *N* = 10 (13%)	24 months	LR
	Stages I-IV	NV				RR: 14.91 (3.67–60.55), *p *= 0.006
Ljumanovic et al. (15) Retrospective	HNSCC; Subsites: oral cavity, Oropharynx, Hypopharynx, Larynx, unknown primary	MRI	Surgery +/− ratiotherapy, radiotherapy, radiochemotherapy	Total *N* = 311; OSCC *N* = 39 (12%)	Mean 34.8 months (7–85 months)	DMFSR
	Stages I-IV	NV				HR: 13.9 (1.4–138.4), *p *= 0.02

nd, no data; ns, not significant; OSCC, oral squamous cell carcinoma; NV, Nodal Volume; LR, locoregional recurrence; HR, hazard ratio; RR, relative risk; DMFSR, distant-metastasis free survival rate.

Two studies investigated not only the oral cavity as tumor site but also other sites such as oropharynx, hypopharynx, and larynx. All the studies focused on squamous cell carcinomas whereas the oropharynx was the most frequent site (15, 16).

The nodal tumor volume was assessed using the planning images before treatment where only one study refers the MRI for examination. The remaining two used the CTs. The nodal volumes were collected either with assistance of semi-automatic/automatic segmentation or by manual segmentation.

Articles assessing the nodal volume referring to the metabolic tumor volume in PET were excluded.

The criteria of a pathologically altered lymph node were central necrosis, which corresponds to a hypo-dense area in the center of the radiographically imaged lymph node, extracapsular spread (ECS), which was defined as radiologically irregular borders (3), and a diameter >10 mm; except in Ljumanovic's et al. (15) study as they chose a diameter >8 mm.

In two studies, patients with all stages of disease were included (I-IV). Note that the clinical staging was made according to the staging system of the UICC 5th edition (1997) (15, 16). Whereby Safi et al. included only patients who were in stage III to IV (according to UICC 7th edition), excluding T4b and patients with lymph node status N2c and N3 (3).

Two out of three studies (3, 16) investigated the locoregional recurrence (LR) as a treatment outcome and one was concerned with the distant-metastasis free survival rate (DMFSR) (15). The proposed cutoff total lymph node volumes were 6.86 cm^3^ (3), 14.00 cm^3^ (16), 5 cm^3^ (15) respectively. Ljumanovic et al. additionally reported the volume of the affected ipsilateral and contralateral nodes separate.

In the univariate and multivariate analysis, all authors came to conclusion, that the volume has a statistically significant association with the LR or DMFSR respectively (Table 1). However regarding the DMFSR, only the contralateral nodes were of significance.

Among the three retrospective cohort-studies, all of them were assessed as low risk of bias. The patient population seemed to be representative, no confounders and the data collection method were shown to be valid.

## 4. Discussion

This systematic review has synthesized the current evidence regarding the prognostic value of nodal tumor volume (NTV) in oral squamous cell carcinoma (OSCC), a topic of increasing relevance in oncology research (11). Our findings suggest that NTV could serve as a valuable prognostic factor in OSCC, offering additional prognostic information beyond traditional N staging. This, in turn, presents an opportunity for more refined risk stratification and individualized therapeutic strategies in OSCC management. While the traditional N staging systems for OSCC primarily focus on the number, size, and laterality of involved lymph nodes, they may not fully capture the complexity and biological variability of nodal disease (3). As OSCC is a highly heterogeneous disease, with substantial interpatient and intratumor heterogeneity, a more nuanced approach to prognostic assessment may be necessary. NTV, by accounting for the three-dimensional nature of the tumor within the lymph node, could provide such an approach (3, 11). Our review found that larger NTV was generally associated with poorer survival outcomes in OSCC. This is consistent with the understanding that larger tumor volume often indicates more advanced disease and a greater tumor burden, which are typically associated with a worse prognosis (17). However, it is noteworthy that this association remained significant in multivariate analyses adjusting for other prognostic factors, suggesting that NTV could serve as an independent prognostic factor. This highlights the potential of NTV to provide unique prognostic information not captured by other factors.

In the comparison between NTV and traditional N staging, NTV often emerged as a more robust predictor of patient outcomes (3, 15, 16). This could be explained by the fact that NTV takes into account the total tumor burden within the lymph nodes, rather than just the number and size of involved nodes. For instance, a patient with multiple small metastases may have the same N stage as a patient with a single large metastasis, but their total tumor burdens (and likely their prognoses) could be quite different. NTV could therefore provide a more accurate reflection of the true extent of nodal disease, allowing for more precise risk stratification and treatment planning.

However, despite the potential advantages of NTV, several challenges need to be addressed to facilitate its clinical application. The lack of standardization in NTV measurement and interpretation is a major issue. The methods for calculating NTV varied widely among the included studies, and there was also substantial variation in the reported ‘cut-off’ values of NTV that were associated with poorer survival outcomes. The included studies were analyzed regarding a statistically significant correlation (*p-*value <0.05) between nodal volume and the respective treatment outcome. The following outcomes were examined: locoregional recurrence and distant-metastasis free survival rate.

Age, sex, N-Stage and lymph node parameters (e.g., central necrosis) were the most common covariates in multivariate analysis. Additionally, Vergeer et al. considered the therapy modality (chemotherapy or radiotherapy) as a covariate (16).

All three studies found a significant association and suggested a cutoff volume of 6.86 cm^3^ and 5 cm^3^ (significant only for contralateral lymph node volume), whereas Vergeer et al. suggests a cutoff volume of 14.00 cm^3^ (3, 16). Interestingly the latter value deviates strongly from the others, although the patient population in all three studies is comparable. An important aspect is the presence of different subsites, which cause different disease progressions. The literature shows that mortality in patients with oropharyngeal cancer (especially in HPV-negative patients) is much higher than in patients with OSCC (18, 19).

These discrepancies underscore the need for more standardized and reliable methods for NTV calculation and interpretation. Future research should aim to develop and validate standardized protocols for NTV measurement, which could be widely adopted in both research and clinical settings.

Furthermore, the use of different imaging modalities for NTV assessment in the included studies raises questions about the optimal imaging technique. While computed tomography (CT) and magnetic resonance imaging (MRI) are commonly used in clinical practice, positron emission tomography (PET) may offer additional information about the metabolic activity of the tumor, which could have prognostic implications. However, most of the articles reviewed for this study used different imaging methods to determine the volume. Two of the included studies (3, 16) used CT as the basis for their measurement and one study (15) employed MRI. A cohort study by Weimar et al. (20) showed that CT and MRI findings reflect the dimension of OSCC very well. Although MRI was slightly more adequate than CT, the difference was not significant. Atudies that used PET-CT to determine the metabolic tumor volume were excluded. This is based on the findings of Venkada et al. that gross tumor volume measured in CT/MRI is not comparable to metabolic tumor volume. Because the GTV delineated at PET tends to be smaller than GTV delineated at CT (21).

Future studies should therefore explore the comparative advantages and limitations of these imaging modalities for NTV assessment.

The potential role of NTV in guiding therapeutic decisions is another important area for future research. If NTV is found to be a robust predictor of survival, it could influence decisions about the extent of neck dissection, the use of adjuvant therapy, and the frequency of follow-up. For instance, patients with high NTV might benefit from more aggressive treatment or closer monitoring, which could potentially improve their survival outcomes. On the other hand, patients with low NTV might be spared unnecessary treatment-related morbidity, improving their quality of life without compromising their prognosis.

Our review provides strong evidence for the prognostic value of NTV in OSCC, it is important to consider these findings in the broader context of OSCC management. OSCC is a complex disease that requires a multifaceted approach to treatment and prognosis. While NTV could be a valuable addition to the prognostic arsenal, it should be used in conjunction with other clinical, pathological, and molecular factors to provide a comprehensive assessment of each patient's prognosis.

In particular, emerging research into the molecular characteristics of OSCC could provide valuable insights into the relationship between NTV and patient outcomes. For instance, certain molecular markers have been associated with aggressive tumor behavior and poor prognosis in OSCC (Zitat: Safi/Ezrin). It would be interesting to explore whether these markers are also associated with larger NTV or whether NTV could modify the prognostic impact of these markers. Such research could further refine our understanding of the prognostic significance of NTV and its interplay with other prognostic factors.

Looking ahead, technological advancements could also facilitate the application of NTV in clinical practice. The development of automated or semi-automated methods for NTV calculation, for instance, could reduce the time and effort required for this process, making it more feasible for routine use. Additionally, the integration of NTV into computerized decision-support systems could assist clinicians in interpreting NTV data and incorporating it into their prognostic assessments and treatment decisions (Segmentierungs-Zitate).

In conclusion, our review suggests that NTV holds promise as a novel prognostic factor in OSCC. By providing a more nuanced understanding of nodal disease, NTV could improve risk stratification, facilitate individualized treatment planning, and ultimately enhance patient outcomes. However, more research is needed to standardize the measurement and interpretation of NTV, elucidate its role in therapeutic decision-making, and integrate it into the broader landscape of OSCC management. As we continue to advance our understanding of OSCC and refine our prognostic tools, we move one step closer to the goal of personalized medicine: providing the right treatment to the right patient at the right time.

## 5. Conclusion

This systematic review indicates that NTV may offer additional prognostic value in OSCC, potentially improving individualized risk assessment and treatment planning. Larger, prospective studies are needed to confirm these findings and establish clear guidelines for the practical application of NTV in clinical settings. Future research should also focus on standardizing NTV measurement and interpretation, exploring the optimal imaging modality for NTV assessment, and investigating the implications of NTV for therapeutic decision-making. With these advancements, NTV could become a valuable tool in the prognostic arsenal for OSCC, leading to more accurate predictions of patient outcomes and more tailored treatment strategies.
